# Morphological Study of Human Cadaveric Livers and Its Clinical Significance

**DOI:** 10.7759/cureus.53873

**Published:** 2024-02-08

**Authors:** Hiteshkumar M Chauhan, Hetal H Modi, Jinesh B Rathod, Himanshu K Prajapati

**Affiliations:** 1 Anatomy, Government Medical College Bhavnagar, Bhavnagar, IND; 2 Anatomy, G.M.E.R.S. Medical College, Himmatnagar, Himmatnagar, IND; 3 Obstetrics and Gynaecology, Tirth Hospital, Himmatnagar, IND; 4 Anatomy, Dr. M. K. Shah Medical College and Research Centre, Ahmedabad, IND

**Keywords:** riedel’s lobe, pons hepatis, papillary process, liver, caudate process

## Abstract

Introduction

The liver is the largest gland in the body and shows wide variation in surface features. Knowledge of external features is essential for radiological investigations and during abdominal surgeries. Morphological variation of the liver should be considered for better patient outcomes. Segmental anatomy has received more attention for segmental resection and transplant surgery. The present study aimed to determine variations in external features of the liver and indicate its clinical importance.

Materials and method

A cross-sectional observational study was conducted on 52 specimens of human cadaveric livers, obtained during routine dissection in the anatomy departments of various medical colleges of Gujarat, India. Livers were examined for their morphology (lobes, notches, fissures, grooves), including their variations; pictures were taken; and results were tabulated.

Result

In the present study, 28 (53.84%) livers were normal in appearance in reference to surfaces, borders, lobes, and fissures. Specifically, 3.84% of livers were found with a very small left lobe, and 1.92% of livers with a large saddle-shaped left lobe. Five (9.61%) livers show the presence of Riedel’s lobe, three (5.76%) livers show deep renal impression, and 13 (25%) livers show grooves on its antero-superior surface. One or two extra fissures were present in 28 livers, either present on the visceral surface of the right lobe, between the caudate process and papillary process of the caudate lobe or quadrate lobe. Pons hepatis was found in 10 (19.22%) livers. Tongue-like projection of the right lobe of the liver was observed in five (9.61%) livers, while an elongated left lobe was observed in three (5.76%) livers.

Conclusion

Livers show wide variations in their surface features. The variations observed in the present study will be of great help to anatomists, radiologists, and surgeons during diagnosis or surgical procedures.

## Introduction

The liver is considered the largest gland in the body, which has both endocrine and exocrine functions [1]. The endocrine function of the liver includes the production of hormones such as 25-hydroxy vitamin D, insulin-like growth factor (IGF-1), etc. and the metabolism of hormones such as thyroid hormone, steroids, and others [2]. The liver develops from the endodermal diverticulum arising at the distal end of the foregut in the middle of the third week of intrauterine life. Epithelial cells of the liver bud interact with vitelline and umbilical veins to form hepatic sinusoids, while hepatic cord cells form parenchyma of the liver [3]. At three months of gestation, the abdominal cavity is filled with liver, and its left and right lobes are almost equal in size. When hematopoietic activity is taken up by the spleen and bone marrow, the left lobe of the liver undergoes some degeneration and becomes smaller than the right lobe [4]. Morphologically, the liver is divided into the right lobe, left lobe, caudate lobe, and quadrate lobe by the fissures. Variations in the morphology of the liver could be due to pressure from the diaphragm, stretch from the coronary ligament, triangular ligament, falciform ligament, ligamentum venosum, and other organs in relation to the liver [5]. Netter classified the liver into seven types according to its shape [6]. Couinaud divided the liver into eight functional segments based on the distribution of branches of the portal vein, hepatic artery, and bile duct with the location of the hepatic vein [1,7].

The liver shows wide variations in the form of agenesis, atrophy, or hypertrophy of its lobe. These variations may be associated with the appearance of extra fissures. Riedel’s lobe is the most common accessory lobe, which projects below the cystic notch of the inferior border, from the right lobe [8].

An elongated left lobe can be associated with chronic abdominal pain, while a small size of the left lobe of the liver may be associated with gastric volvulus, and problems in the right lobe of the liver lead to hypertension [9]. Accessory fissures of the liver can be falsely diagnosed as liver injury, and small accessory lobes can be diagnosed as lymph nodes. Thus, detailed knowledge of morphological features is required to prevent wrong radiological interpretations and surgical procedures [4,10-12].

Aim of the study

Many studies have been done on segments of the liver based on the distribution of the structure of portal triad and hepatic vein tributaries, which is required for transplantation of the liver and other surgical procedures. However external features of the liver are equally important for radiological diagnosis, laparoscopic surgeries, and other major transplant surgeries. The present study aimed to (1) study the morphology of the various lobes of the liver and the presence of any accessory lobe of the liver, (2) study the morphology of fissures and note the presence of any accessory fissures, and (3) determine the presence of pons hepatis bridges along the groove for ligamentum teres or groove for inferior vena cava and its clinical importance.

## Materials and methods

A cross-sectional observational study was conducted in September 2023 on 52 human adult cadaveric livers obtained during routine dissection in the anatomy department of various medical colleges in Gujarat, India. During routine anatomy dissection of the abdomen, an incision was put in the midline from the xiphoid process to pubic symphysis and extended on each side laterally from the xiphoid process, umbilicus, and pubic symphysis to reflect the skin in four flaps. The superficial fascia, abdominal muscles, and parietal peritoneum were incised to expose the liver. The liver was pulled downwards, and the anterior layers of the coronary and left triangular ligaments were divided. The inferior vena cava (IVC) was identified between the liver and diaphragm, and separated from the liver. If the IVC was found deeply buried in the liver, a segment of it was removed along with the liver. The structures in the porta hepatis were exposed and cut close to the porta hepatis. The liver was separated from all the peritoneal ligaments and folds of the liver and removed from the cadaver as per Cunningham's Manual of Practical Anatomy [13]. The specimens were preserved in a 10% solution of formalin. All the normal external features such as various borders, surfaces, and lobes were identified, and the specimens showing any evidence of disease, surgery, or damage were excluded from the study.

These livers were observed for any surface variations in the form of extra fissures, extra notches, variations in the right and left lobes, and variations in the caudate and quadrate lobes. Livers were observed for their fissure for ligamentum venosum, fissures for ligamentum teres, and porta hepatis.

**Figure 1:**
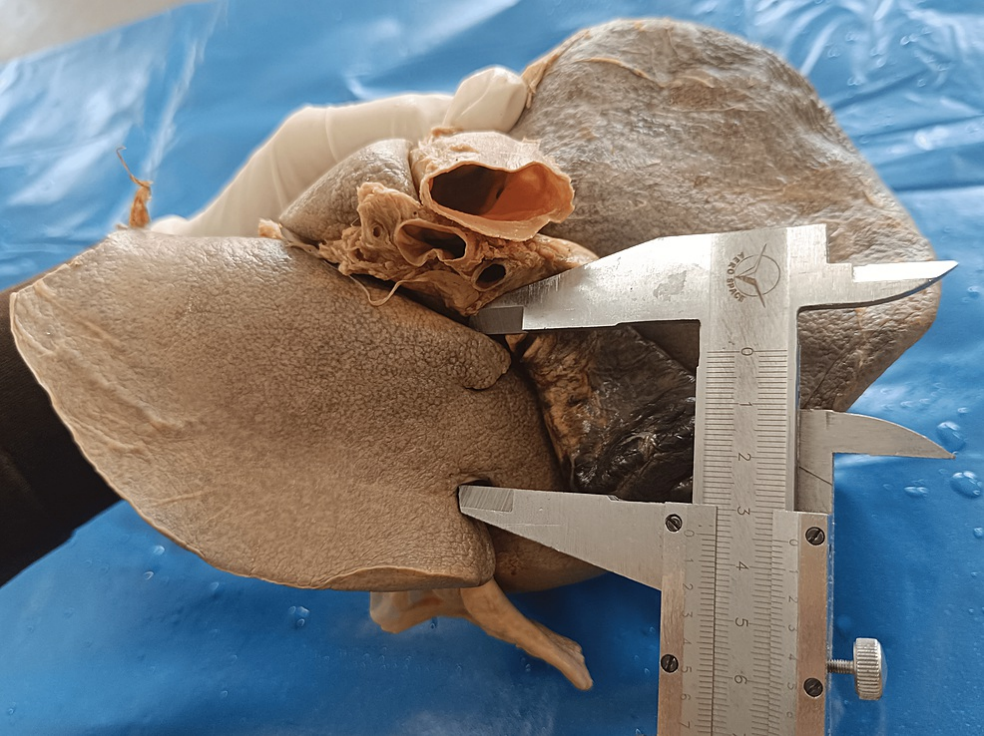
Measurement of pons hepatis with a vernier caliper

They were also observed for the presence of pons hepatis (a band of liver tissue bridging the groove for IVC or groove for ligamentum teres), and its site was noted. Pons hepatis that bridges the IVC was observed as completely bridging or partially covering IVC. Pons hepatis that covers the groove for ligamentum teres was observed and classified in open or closed type from its length, from transverse fissure to the anterior margin of pons hepatis. Measurements of pons hepatis were taken with the help of the vernier caliper (Figure 1).

Photographs of the variations were taken, and results were tabulated. Livers were classified according to Netter’s classification. A liver with all normal features was considered as type 1, a very small left lobe with deep costal impression as type 2, complete atrophy of the left lobe as type 3, a transverse saddle-like liver with a relatively large left lobe as type 4, a tongue-like process of the right lobe as type 5, a liver that had deep renal impression with corset constriction as type 6, and a liver that had diaphragmatic grooves as type 7 [5].

## Results

**Figure 2:**
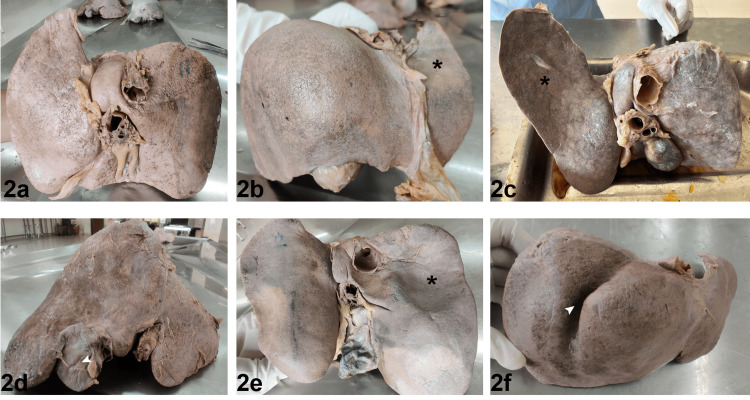
Liver categorized as per Netter’s anatomical classification 2a. Netter’s type 1, 2b. Netter’s type 2 (* shows a very small left lobe), 2c. Netter’s type 4 (* shows a saddle-like left lobe), 2d. Netter’s type 5 (a white arrowhead shows Reidel’s lobe), 2e. Netter’s type 6 (* shows deep renal impression), 2f. Netter’s type 7 (a white arrowhead shows a diaphragmatic groove)

In the present study, 52 liver specimens were observed for morphological variations. The livers were classified according to Netter’s classification. Out of 52 livers examined, 54% were of type 1 normal, and 25% of the type 7 variety had diaphragmatic grooves, while none found of the type 3 variety in reference to surfaces, borders, lobes, and fissures, as per Netter’s classification (Figure 2, Table 1).

**Table 1:** Prevalence based on Netter’s anatomical classification (n=52)

Netter’s type	Gross description	Number of liver (percentage)
Type 1	Normal	28 (53.84%)
Type 2	Very small left lobe, deep costal impression	02 (3.84%)
Type 3	Complete atrophy of the left lobe	00
Type 4	Transverse saddle-like liver, relatively large left lobe	01 (1.92%)
Type 5	Tongue-like process of right lobe	05 (9.61%)
Type 6	Deep renal impression and corset constriction	03 (5.76%)
Type 7	Diaphragmatic grooves	13 (25%)

**Figure 3:**
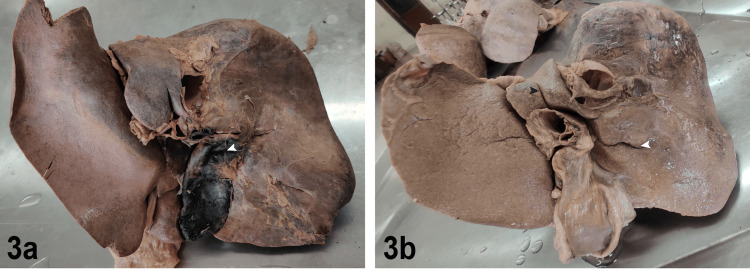
Fissures on various surfaces of the liver 3a. Fissure on the quadrate lobe (white arrowhead), 3b. Fissure separating the caudate process (black arrowhead) and fissure on the inferior surface of the right lobe (white arrowhead)

In observations, we found one or two extra fissures in 28 livers either present on the visceral surface of the right lobe (called Rouvieres sulcus and is of surgical importance during laparoscopic cholecystectomy), between the caudate process and papillary process of the caudate lobe (bicornuate caudate lobe), or fissure present on the quadrate lobe dividing it into two halves (Figure 3, Table 2).

**Table 2:** Prevalence of fissures on various surfaces of the liver

Location of extra fissure	Number of liver (percentage)
On the posterior aspect of the right lobe	09 (17.30%)
Fissure between the caudate process and papillary process	13 (25.00%)
Fissure on the quadrate lobe	06 (11.53%)

**Figure 4:**
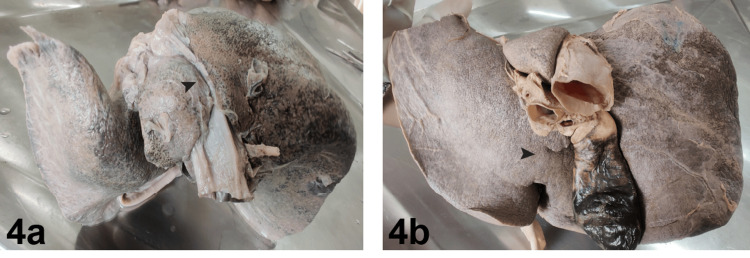
Types of pons hepatis 4a. Pons hepatis bridging the groove for the inferior vena cava (black arrowhead), 4b. Pons hepatis bridging the groove for the ligamentum teres (black arrowhead)

Pons hepatis bridging the groove for inferior vena cava was found in 9.61% of livers, and pons hepatis bridging the groove for ligamentum teres was also found in 9.61% of livers (Figure 4).

**Figure 5:**
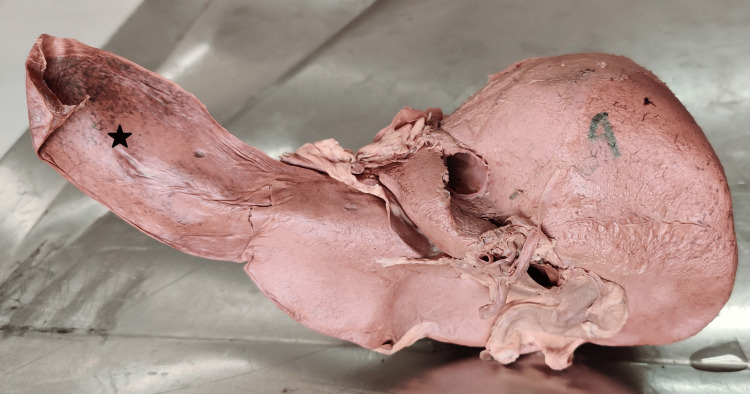
Tongue-like elongation of the left lobe of the liver * shows an elongated left lobe of the liver

Tongue-like projections of the right lobe of the liver (Riedel’s lobe) were observed in 9.61% of livers, while an elongated left lobe was observed in 5.76% of livers (Figure 5).

Right lobe

The right lobe appears normal in 28 livers with no other impressions. Nine livers showed fissures on the posterior surface, five livers had tongue-like projections, 13 livers had diaphragmatic grooves on the anterosuperior surface, and three livers showed deep renal impressions.

Left lobe

Two livers had a small atrophied left lobe, three livers showed an elongated left lobe, and one liver showed a large saddle-shaped left lobe.

Caudate lobe

It was of medium size in most of the livers with a rectangular shape. In some livers, it was of pear shape and some with triangular shape. Some livers showed the elongated papillary process. Thirteen livers had fissures separating the caudate process and papillary process giving a bicornuate appearance of the caudate lobe. Pons hepatis bridging the groove for the inferior vena cava was present in five livers, which completely covers the groove for IVC.

Quadrate lobe

It was of medium size and quadrangular in shape in most livers. However, six livers showed the presence of an accessory quadrate lobe separated by a fissure. Pons hepatis bridging fissures for the ligamentum teres were present in five livers. Out of these, three livers had the close type of pons hepatis having a length of more than 2 cm, and two livers had an open type of pons hepatis having a length of less than 2 cm.

## Discussion

Morphological variations in the liver found on autopsy or anatomy dissection remain undiagnosed due to its asymptomatic nature. A comparison of the morphological variations of the liver in other studies with the present study has been tabulated (Table 3).

**Table 3:** Comparison of morphological variations of the liver in various studies with the present study IVC = Inferior vena cava

Morphological variations	Neginhal et al. [14]	Singh et al. [5]	Mansur et al. [15]	Sharma et al. [16]	Singh [17]	Present study
Accessory fissure	-	-	23 (32.86%)	-	16 (32%)	27 (51.92%)
Extra fissure on the right lobe	10 (20%)	36 (51.43)	-	13 (17.33%)	-	09 (17.30%)
Extra fissure on caudate lobe	04 (8%)	19 (27.14)	-	05 (6.66%)	06 (12%)	13 (25%)
Extra fissure on quadrate lobe	11 (22%)	23 (32.86)	-	04 (5.33%)	-	06 (11.53%)
Pons hepatis bridging groove for IVC	-	16 (22.86%)	-	-	-	05 (9.61%)
Pons hepatis bridging fissure for ligamentum teres	08%	-	11 (15.71%)	02 (2.66%)	08 (16%)	05 (9.61%)
Riedel’s lobe	01 (2%)	-	-	03 (4%)	-	05 (9.61%)
Elongated left lobe (Beaver's lobe)	04%	09 (12.86%)	05 (7.14%)	07 (9.33%)	07 (14%)	03 (5.76%)
Diaphragmatic grooves	03 (6%)	08 (11.43%)	-	02 (1.5%)	07 (14%)	13 (25%)
Deep renal impression	03 (6%)	-	-	15 (20%)	-	03 (5.76%)

Right lobe

The right lobe is anatomically the largest lobe of the liver, six times larger than the left lobe, but physiologically both lobes are almost equal in size [17]. It is situated under the right dome of the diaphragm. The right lobe is separated from the left lobe of the liver by a fissure for the ligamentum venosum on the posterior surface, ligamentum teres on the inferior surface, and anteriorly by the falciform ligament. Livers show accessory fissure on the right lobe in Neginhal et al.'s [14] study (20%), Cawich et al.'s [18] study (19, 31.7%), Mansur et al.'s [15] study (23, 32.86%), Singh et al.'s [5] study (36, 51.43%), and Sharma et al.'s [16] study (13, 17.33%). In the present study, nine livers have fissures on the posterior aspect of the right lobe of the liver, suggesting that accessory fissures are more common in the right lobe.

Neginhal et al. [14] found one (2%) liver, Sharma et al. [16] found three (4%) livers, and five (9.61%) livers have Riedel’s lobe in the present study. Neginhal et al.'s [14] study found three (6%) livers with a deep renal impression, 15 (20%) livers in Sharma et al.'s [16] study, and three (5.76%) livers in the present study. Livers that had diaphragmatic grooves on the anterosuperior surface are found: three (6%) livers in Neginhal et al.'s [14] study, eight (11.43%) livers in Singh et al.'s [5] study, two (1.5%) livers in Sharma et al.'s [16] study, seven (14%) livers in Singh's [17] study, and 13 (25%) livers in the present study. This shows a higher prevalence of diaphragmatic grooves in the present study.

Left lobe

The left lobe is anatomically smaller than the right lobe. The left lobe extends towards the fundus of the stomach and helps keep the stomach in position. If the left lobe is smaller in size, it can be associated with gastric volvulus, and if it is elongated or large, it can be associated with abnormal abdominal or epigastric pain. In the elongated left lobe, Bismuth-Couinaud segment-2 is commonly enlarged [4]. Elongated left lobe are found in Neginhal et al.'s [14] study (two livers, 4%), Sharma et al.'s [16] study (seven livers, 9.33%), and Dawani et al.'s [17] (seven livers, 14%). In the present study, three (5.76%) livers show an elongated left lobe. Anbumani et al.'s [9] study found that 3.30% of the liver shows a small left lobe, while, in the present study, two (3.84%) livers have a small left lobe. The present study shows one (1.92%) liver with a saddle-shaped large left lobe.

Caudate lobe

It is situated on the posterior surface of the liver between the fissure for the ligamentum venosum and the groove for the inferior vena cava. It has an elongation caudate process from the right lower corner and a papillary process from the left lower corner of the lobe.

The pons hepatis is a segment of hepatic tissue connecting the quadrate lobe to the left lobe over the ligamentum teres fissure, or the pons hepatis refers to hepatic tissue that surrounds the inferior vena cava [19], which has a wide range in morphology. The pons hepatis bridging the inferior vena cava was found in Anbumani et al.'s [9] study (6.66% of livers), Chin et al.'s [19] study (12 livers, 36.36%), and Reddy et al.'s [20] study (4-30% of livers). In the present study, pons hepatis bridging the inferior vena cava was present in five (9.61%) livers.

Extra fissures on the caudate lobe are found in Neginhal et al.'s [14] study (four livers, 8%), Singh et al.'s [5] study (19 livers, 27.14), Sharma et al.'s [16] study (five livers, 6.66%), and Dawani et al.'s [17] study (six livers, 12%). In the present study, 13 (25%) livers show fissures on the caudate lobe along with the bicornuate appearance of the caudate lobe, coinciding with that of Singh et al.'s study [5], but higher prevalence than other studies.

Quadrate lobe

It is present below the porta hepatis, between the fissure for the ligamentum teres and the groove for the inferior vena cava, and on the visceral surface of the liver. Extra fissure on the quadrate lobe was noted by Neginhal et al. [14] in 22% of livers, Singh et al. [5] in 32.86% of livers, and Sharma et al. [16] in 5.33% livers. In the present study, extra fissure on the quadrate lobe was reported in 11.53% of livers, which lies within the range of variation of earlier studies.

The pons hepatis bridging fissures for the ligamentum teres was noted by Singh et al. [5] in 16 (22.86%) livers, Anbumani et al. [9] in three (10%) livers, Neginhal et al. [13] in 0.8% of livers, Mansur et al. [15] in 11 (15.71%) livers, Sharma et al. [16] in two (2.66%) livers, and Singh [17] in eight (16%) livers. In the present study, the pons hepatis bridging the groove for the ligamentum teres was found in five (9.61%) livers, among which three were of a close type and two were of open type, which coincides with other studies.

This study was done in livers isolated from the embalmed cadavers because, with formalin, the liver may get hardened and show exaggeration of some impression. This study can be extended further in collaboration with radiological studies of living human beings to know the impact or significance of these variations.

## Conclusions

Large variations were found in the surface features of the liver in the present study. These variations will help anatomists understand the morphological variations of the liver and guide radiologists to do proper diagnosis without the confusion of fissures, injuries, accessory lobes, metastatic deposits, or enlarged lymph nodes. These variations will help surgeons plan surgeries accordingly. This study can be extended further in collaboration with radiological studies of living human beings to know the impact or significance of these variations.
